# Evaluating the Predictive Value of HOMA-IR in Gestational Diabetes: A Case–Control Study from Romania

**DOI:** 10.3390/diagnostics15131704

**Published:** 2025-07-03

**Authors:** Ait el Haj Iman, Cristina Onel, Gheorghe Furau, Cristian Furau, Roxana Furau, Mihai Lucan, Mircea Sandor, Liliana Sachelarie, Anca Huniadi

**Affiliations:** 1 Department of Clinical Disciplines, Faculty of Medicine and Pharmacy, University of Oradea, 1st December Square 10, 410073 Oradea, Romania; ait.iman@csud.uoradea.ro (A.e.H.I.); luncan.simionmihai@student.uoradea.ro (M.L.); ahuniadi@uoradea.ro (A.H.); 2 Department of Obstetrics and Gynecology, Western University “Vasile Goldis” of Arad, 310025 Arad, Romania; onel.cristina@uvvg.ro (C.O.); furau.gheorghe@uvvg.ro (G.F.); 3 Life Sciences Department, Western University “Vasile Goldis” of Arad, 310025 Arad, Romania; furau.cristian@uvvg.ro; 4 Department of General Medicine, Faculty of Medicine, Western University “Vasile Goldis” of Arad, 310414 Arad, Romania; furau.roxana@uvvg.ro; 5 Department of Preclinical Discipline, Apollonia University, 700511 Iasi, Romania; 6 Calla—Infertility Diagnostic and Treatment Center, Constantin A. Rosetti Street, 410103 Oradea, Romania; 7 Pelican Clinical Hospital, Corneliu Coposu Street 2, 410450 Oradea, Romania

**Keywords:** gestational diabetes mellitus (GDM), insulin resistance, HOMA-IR, body mass index (BMI)

## Abstract

**Background/Objectives:** Gestational diabetes mellitus (GDM) is a common metabolic disorder during pregnancy, associated with increased risks for both maternal and fetal complications. Insulin resistance plays a central role in its pathophysiology. This study aimed to evaluate the predictive value of the Homeostatic Model Assessment for Insulin Resistance (HOMA-IR) in diagnosing GDM and to explore its correlation with clinical and anthropometric parameters in a Romanian population. **Methods:** A retrospective case–control study was conducted on 320 pregnant women between 24 and 28 weeks of gestation. Based on ADA criteria, participants were divided into 160 with GDM and 160 controls, matched by age and gestational week. Fasting glucose, insulin, BMI, and blood pressure were assessed. HOMA-IR and HOMA-β were calculated. Statistical analyses included *t*-tests, Pearson correlation, and logistic regression. **Results**: HOMA-IR was significantly higher in the GDM group (2.9 vs. 1.8; *p* < 0.001). It correlated with fasting insulin (r = 0.85, *p* < 0.001), fasting glucose (r = 0.65, *p* < 0.001), BMI (r = 0.60, *p* < 0.001), and systolic blood pressure (r = 0.42, *p* < 0.001). Logistic regression identified HOMA-IR as an independent predictor of GDM (OR = 2.4, 95% CI: 1.6–3.5, *p* < 0.001), along with BMI (*p* = 0.01) and maternal age (*p* = 0.05). **Conclusions:** HOMA-IR is significantly associated with GDM and may enhance mid-gestational risk assessment when combined with clinical and anthropometric measures. Further studies are needed to validate its predictive accuracy in broader populations.

## 1. Introduction

Gestational diabetes mellitus (GDM) is one of the most common metabolic disorders encountered during pregnancy, with increasing prevalence worldwide due to rising rates of obesity and delayed childbearing [1,2]. GDM is associated with a wide range of adverse maternal and fetal outcomes, including hypertensive complications, macrosomia, neonatal hypoglycemia, and long-term risk of type 2 diabetes mellitus (T2DM) in both mother and child [3,4].

A key underlying mechanism in the development of GDM is insulin resistance, which usually increases in late pregnancy to support fetal growth but can become pathologic when exaggerated or superimposed on pre-existing metabolic risk [5,6]. In this context, early identification of women at increased risk for GDM is crucial for timely intervention and improved outcomes.

The Homeostatic Model Assessment of Insulin Resistance (HOMA-IR) is a simple, non-invasive surrogate marker derived from fasting plasma glucose and insulin levels. It has been widely used in clinical and research settings due to its affordability and ease of calculation [7]. HOMA-IR has shown a strong correlation with the euglycemic–hyperinsulinemic clamp, which remains the gold standard for insulin sensitivity [8,9]. As such, HOMA-IR has gained increasing attention as a potential early marker of metabolic dysfunction in pregnancy [10].

Nevertheless, the predictive accuracy of HOMA-IR for GDM remains inconsistent, with its performance varying based on maternal characteristics, testing timing, and population-specific factors [11,12]. While numerous studies have investigated HOMA-IR in pregnancy, most originate from North America and Asia. Research from Eastern European populations is sparse, despite this region’s unique metabolic and socio-demographic profiles. This gap highlights the importance of validating such markers in diverse settings to ensure clinical relevance. Although several diagnostic criteria for GDM exist, there is still no universally accepted biomarker for early prediction, and current approaches often rely on late second-trimester glucose tolerance testing. Identifying reliable, non-invasive, and early-pregnancy predictors remains a clinical priority.

Currently, gestational diabetes is typically diagnosed by the oral glucose tolerance test (OGTT) performed in the second trimester [8]. However, this method has significant limitations, including delayed diagnosis and patient burden due to multiple blood draws and strict testing conditions. Therefore, there is growing interest in identifying early, non-invasive biomarkers in the first trimester. This could allow timely risk stratification and improved monitoring, ultimately reducing adverse outcomes associated with late diagnosis.

Beyond biochemical indices, anthropometric parameters such as body mass index (BMI) are widely recognized predictors of insulin resistance and GDM [13]. These markers, reflecting both general and visceral adiposity, are easy to obtain during routine prenatal visits and may enhance the utility of HOMA-IR in composite risk models. In addition, clinical variables such as maternal age and blood pressure have been implicated in the development of GDM, although their predictive value may be more context-dependent [14,15].

Given GDM’s multifactorial etiology, there is growing interest in integrating biochemical, anthropometric, and clinical variables into risk stratification models tailored to specific populations. These models can improve early detection, personalize monitoring strategies, and inform targeted interventions.

The present study aims to assess the association between HOMA-IR and GDM in a Romanian cohort and to evaluate its relationship with BMI, maternal age, and blood pressure. By contributing population-specific data from an underrepresented region, this research supports the development of more inclusive and applicable predictive tools in prenatal care.

## 2. Materials and Methods

### 2.1. Study Design and Variables

This retrospective case–control study was conducted over a 12-month period (January–December 2023) at the Calla Center for Obstetrics and Gynecology in Oradea, Romania. The primary objective was to evaluate the association between insulin resistance, assessed through the Homeostatic Model Assessment for Insulin Resistance (HOMA-IR), and the presence of gestational diabetes mellitus (GDM), as well as to analyze its correlation with selected anthropometric and clinical parameters. Consecutive sampling was used to identify all eligible pregnant women who presented at the clinic during this timeframe. Approximately 2100 patients were evaluated, and women were included based on predefined inclusion and exclusion criteria and then retrospectively grouped into GDM and non-GDM cohorts, matched by maternal age and gestational week. No formal a priori sample size calculation was conducted. However, a post hoc power analysis based on the observed difference in HOMA-IR values between groups (mean difference = 1.1, standard deviation ≈ 1.0) indicated that the study had a statistical power exceeding 95% to detect a significant difference at an alpha level of 0.01. This suggests that the sample size was adequate for the primary outcome of interest.

All participants were evaluated during routine prenatal visits between 24 and 28 weeks of gestation. The diagnosis of GDM was established using the one-step 75 g oral glucose tolerance test (OGTT), interpreted according to the American Diabetes Association (ADA) and the International Association of Diabetes and Pregnancy Study Groups (IADPSG) criteria. Based on these results, participants were divided into two groups: Group A, consisting of 160 women diagnosed with GDM, and Group B, consisting of 160 non-GDM pregnant women, matched by maternal age and gestational age.

Exclusion criteria included pre-existing type 1 or type 2 diabetes, a prior history of gestational diabetes mellitus (GDM), multiple pregnancies, polycystic ovary syndrome (PCOS), known chronic systemic diseases (such as autoimmune, hepatic, renal, or cardiovascular conditions), and the use of medications known to affect glucose metabolism, including corticosteroids.

Blood samples were collected in the morning after an overnight fast of at least 8 h, between 24 and 28 weeks of gestation. Fasting plasma glucose was measured using an enzymatic colorimetric method (glucose oxidase) on the Roche Cobas c111 analyzer. Fasting insulin levels were assessed via chemiluminescent immunoassay on the Roche Elecsys 2010 platform. All assays followed the manufacturer’s protocols and adhered to internal laboratory quality standards. The intra-assay coefficients of variation were below 3% for glucose and 6% for insulin.

Blood pressure was measured using a standard automated oscillometric sphygmomanometer, with the patient seated and after a 5-min rest. Anthropometric measurements included maternal age, body mass index (BMI), and blood pressure. BMI was calculated from the measured weight and height.

Blood pressure was recorded as the average of two measurements taken five minutes apart, using a calibrated automatic sphygmomanometer. Maternal age was obtained from clinical records and verified during consultation.

HOMA-IR and HOMA-β indices were calculated from fasting glucose and insulin concentrations using the formulas described by Matthews et al. [2], widely accepted in clinical and research settings to estimate insulin resistance and β-cell function, respectively. All clinical assessments and laboratory procedures were standardized to ensure consistency and reproducibility.

### 2.2. Statistical Analysis

Statistical analysis was performed using IBM SPSS Statistics version 26.0 (IBM Corp., Armonk, NY, USA). Descriptive statistics (mean ± SD or median and interquartile range) were calculated for all variables. The differences between the two groups were assessed using Student’s *t*-test or the Mann–Whitney U-test for continuous variables and the chi-square test for categorical variables.

Pearson correlation analysis was used to assess the relationships between HOMA-IR and continuous clinical or anthropometric variables. The strength of the correlation coefficient (r) was interpreted as follows: very weak (0.00–0.19), weak (0.20–0.39), moderate (0.40–0.59), strong (0.60–0.79), and very strong (0.80–1.00).

A multivariable logistic regression model was constructed to identify independent predictors of GDM. Variables that were statistically significant in univariate analysis (*p* < 0.05) or considered clinically relevant were included in the model: age, BMI, fasting glucose, and HOMA-IR. Statistical significance was set at *p* < 0.01 for all analyses.

## 3. Results

A total of 320 pregnant women were included in the final analysis, with 160 diagnosed with GDM and 160 non-GDM controls matched by age and gestational week.

**Figure 1:**
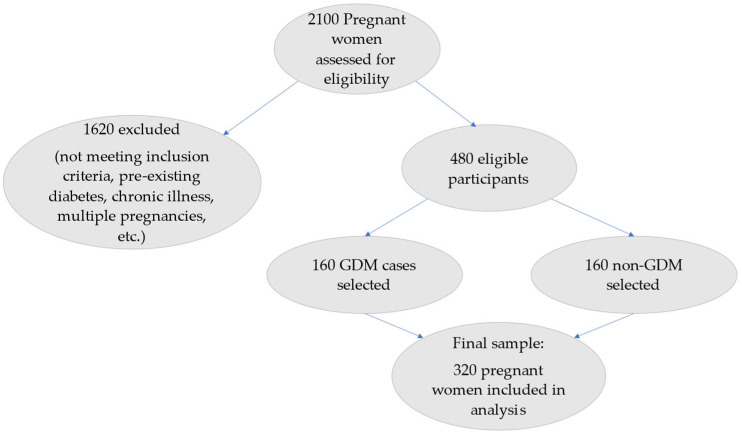
Flowchart showing patient selection and final study sample.

A flow diagram of participant selection and grouping is presented in Figure 1.

### 3.1. Demographic and Clinical Characteristics

Table 1 presents the demographic and clinical characteristics of the two study groups: women diagnosed with gestational diabetes (*n* = 160) and those without GDM (*n* = 160). Statistically significant differences between the groups (*p* < 0.01) are highlighted.

Women diagnosed with gestational diabetes mellitus (GDM) exhibited significantly different clinical profiles compared to those without GDM. The GDM group had a higher mean age (30.1 ± 4.8 vs. 28.5 ± 4.2 years, *p* = 0.02) and a higher body mass index (27.5 ± 3.8 vs. 24.3 ± 3.1 kg/m^2^, *p* < 0.001).

In terms of metabolic parameters, women with GDM had significantly elevated fasting glucose levels (95.6 ± 12.3 vs. 85.2 ± 10.5 mg/dL, *p* < 0.001), fasting insulin levels (12.4 ± 4.5 vs. 8.5 ± 3.2 µU/mL, *p* < 0.001), and higher HOMA-IR scores (2.9 ± 1.1 vs. 1.8 ± 0.7, *p* < 0.001). HOMA-β was also significantly increased in the GDM group (180.5 ± 60.7 vs. 150.2 ± 50.3, *p* = 0.01). Systolic and diastolic blood pressure values were also higher among women with GDM (120/80 mmHg vs. 110/70 mmHg, *p* = 0.03). All parameters were measured during the 24th to 28th weeks of gestation to ensure comparability between the two groups.

### 3.2. Pearson Correlation Coefficients (r) and p-Values 

Table 2 presents Pearson correlation coefficients (r) and *p*-values for the relationships between the HOMA-IR index and other clinical and demographic variables. The coefficients provide information about the direction and strength of the relationships, while the *p*-values indicate statistical significance.

Table 2 presents the Pearson correlation coefficients (r) and associated *p*-values for the relationships between HOMA-IR and selected clinical and demographic variables. As expected, HOMA-IR showed a strong positive correlation with fasting insulin (r = 0.85, *p* < 0.001) and a strong correlation with fasting glucose (r = 0.65, *p* < 0.001), both of which are components of its calculation formula. These associations confirm internal consistency but do not provide an independent association with GDM diagnosis.

In addition to fasting glucose, HOMA-IR was also correlated with post-load plasma glucose levels. Pearson correlation analysis revealed an influential association with fasting glucose (r = 0.96), a moderate correlation with 1 h post-load glucose (r = 0.69), and a weaker correlation with 2-h post-load glucose (r = 0.54).

Moderate correlations were observed between HOMA-IR and BMI (r = 0.60, *p* < 0.001). These findings support the link between insulin resistance, obesity-related anthropometric parameters, and compensatory beta-cell activity.

Weaker correlations were identified between HOMA-IR and maternal age (r = 0.30, *p* = 0.04) and blood pressure (r = 0.25, *p* = 0.05). While statistically significant, these associations were weak and should be interpreted cautiously, particularly considering the study’s cross-sectional nature and the potential influence of gestational physiological changes.

The correlation analysis highlights expected associations between HOMA-IR and key metabolic markers while suggesting potential relationships with anthropometric variables. However, due to the study design, these correlations should not be interpreted as evidence of causality.

### 3.3. Logistic Regression Analysis for Predictors of Gestational Diabetes

Table 3 presents the logistic regression analysis results, which determine the predictive power of variables for gestational diabetes. Odds ratios (ORs), confidence intervals (95% CI), and *p*-values, which indicate the statistical significance of each variable, are provided.

A multivariable logistic regression analysis was performed to identify independent predictors of gestational diabetes mellitus (GDM). The model included HOMA-IR, body mass index (BMI), and maternal age as potential covariates.

HOMA-IR emerged as the strongest predictor of GDM, with an odds ratio (OR) of 2.4 (95% CI: 1.6–3.5, *p* < 0.001), indicating that higher insulin resistance significantly increased the likelihood of GDM diagnosis. BMI also remained significant, with ORs of 1.2 (95% CI: 1.1–1.4, *p* = 0.01), respectively, suggesting that general and central obesity contribute independently to GDM risk. Maternal age was marginally associated with GDM (OR = 1.1, 95% CI: 1.0–1.2, *p* = 0.05).

These findings highlight the multifactorial nature of GDM and support using combined clinical and metabolic markers, such as HOMA-IR, and BMI, to improve early identification and risk stratification in pregnancy.

### 3.4. Metabolic Score Analysis

Table 4 presents the distribution of gestational diabetes mellitus (GDM) cases according to cumulative metabolic score categories ranging from 0 to 4. 

Each point in the score corresponds to a specific metabolic risk factor: BMI ≥ 25 kg/m^2^, fasting glucose ≥ 90 mg/dL, and HOMA-IR ≥ 2.5. The total score reflects each patient’s coexisting metabolic alterations during evaluation.

To assess the combined impact of multiple metabolic risk factors on the likelihood of developing gestational diabetes mellitus (GDM), a cumulative metabolic score was constructed, ranging from 0 to 4. One point was assigned for each of the following criteria: body mass index (BMI) ≥ 25 kg/m^2^, fasting glucose ≥ 90 mg/dL, and HOMA-IR ≥ 2.5. The total score thus reflected the number of concurrent metabolic risk features present during evaluation.

Table 4 presents the distribution of GDM cases across the different score categories. A clear upward trend was observed in GDM prevalence with increasing scores: only 10% of women with a score of 0 had GDM, compared to 25% with a score of 1, 50% with a score of 2, 75% with a score of 3, and 92% among those with the maximum score of 4. This gradient highlights the cumulative relationship between metabolic alterations and GDM risk.

## 4. Discussion 

This study reinforces the critical role of insulin resistance in the pathophysiology of gestational diabetes mellitus (GDM), with HOMA-IR emerging as a significant metabolic indicator in the Romanian cohort. The observed elevation in HOMA-IR in the GDM group supports its predictive potential, aligning with prior research indicating that elevated insulin resistance often precedes glucose dysregulation in pregnancy [5,11,16].

The strong correlation between HOMA-IR and fasting insulin (r = 0.85) and glucose (r = 0.65) observed in our study reflects the expected mathematical interdependence, yet it also underscores their concurrent pathophysiological elevation in GDM, as highlighted by Duo et al. [10]. Significantly, HOMA-IR also correlated moderately with BMI (r = 0.60), confirming obesity as a major contributor to insulin resistance. This finding is consistent with evidence from Catalano and Shankar [17] and is echoed in recent meta-analyses showing BMI as a key modifier of GDM risk [18,19].

Our results further demonstrated a modest correlation between HOMA-IR and maternal age (r = 0.30), aligning with studies by Li et al. [20], who showed that advancing maternal age is independently associated with a higher incidence of GDM, possibly due to age-related decline in insulin sensitivity. Similarly, the weak association with blood pressure (r = 0.25) may reflect underlying endothelial dysfunction or subclinical inflammation, mechanisms previously associated with insulin resistance in pregnancy [21,22].

The logistic regression model substantiated the independent predictive value of HOMA-IR (OR = 2.4), BMI (OR = 1.2), and maternal age (OR = 1.1). These findings corroborate evidence that combined anthropometric and biochemical risk factors provide superior predictive accuracy over isolated metrics [23,24]. Bano et al. and Kim et al. emphasized that insulin resistance markers like HOMA-IR perform best when interpreted alongside clinical variables such as BMI or waist circumference [17,21].

A key contribution of this study is the implementation of a cumulative metabolic risk score, combining HOMA-IR, fasting glucose, and BMI. The clear dose–response pattern observed from 10% GDM prevalence in women scoring 0 to 92% in those scoring 4 demonstrates the additive nature of these metabolic disruptions. Similar cumulative scoring models have proven effective in the prior literature. Riskin-Mashiah et al. [22] and Nerenberg et al. [23] proposed multivariate models that included maternal weight, glucose levels, and inflammatory markers to refine early GDM prediction.

Furthermore, our cumulative score provides practical applicability in clinical settings. Unlike OGTT, which is time-consuming and invasive, risk scores derived from routinely available parameters (e.g., BMI, fasting labs) allow for earlier risk stratification. This concept aligns with the work of Inthavong et al. [25], who advocated for simplified biochemical prediction algorithms in mid-pregnancy, and with the growing body of literature advocating for first-trimester screening tools [8,9].

Regarding the pathophysiological interpretation, the elevated HOMA-β in the GDM group suggests a compensatory pancreatic β-cell response to rising insulin resistance, a phenomenon well-documented in the early stages of metabolic dysregulation in pregnancy [3,26]. However, this compensation may eventually fail, leading to impaired glucose tolerance.

Despite its utility, the limitations of HOMA-IR should not be overlooked. Balkaş and Çelen [15] emphasized that its reliability can vary with gestational timing and population characteristics. Our findings suggest that HOMA-IR alone may not suffice for universal screening, but it gains value when integrated into multifactorial models, especially in regions like Eastern Europe where specific metabolic and demographic features differ from Western cohorts [27,28].

Additionally, the implications of GDM extend beyond pregnancy. Numerous studies have associated GDM with increased long-term risk of type 2 diabetes and cardiometabolic disease in mothers [29,30], as well as adverse fetal programming effects that predispose offspring to obesity and insulin resistance later in life [6,31]. As such, early identification of at-risk women is paramount, not only to prevent immediate obstetric complications but also to mitigate long-term intergenerational risks.

Lastly, lifestyle interventions targeting modifiable risk factors such as BMI and dietary patterns have shown promise in preventing GDM onset. The Cochrane review by Brown et al. [32] and the study by Lin et al. [33] provide compelling evidence for the effectiveness of early nutrition and exercise programs in reducing GDM incidence. Integrating HOMA-IR and other risk factors into screening protocols could facilitate early enrollment in such preventive strategies.

This study has several limitations that should be considered. First, while helpful in identifying associations, the retrospective case–control design does not allow for causal inference. Moreover, such designs may overestimate the strength of associations due to artificial differences between selected groups, which may not accurately reflect real-world population distributions. Second, although the groups were matched for maternal and gestational age, residual confounding factors such as lifestyle, diet, and family history were not accounted for. Third, HOMA-IR was measured only once during mid-gestation; repeated assessments throughout pregnancy would offer a more comprehensive view of insulin resistance dynamics. Nonetheless, these findings contribute valuable data to understanding insulin resistance and GDM risk in Eastern European populations and lay the groundwork for future prospective research.

## 5. Conclusions

Our findings indicate that HOMA-IR has potential as a component of mid-gestational risk assessment for gestational diabetes mellitus (GDM). While HOMA-IR alone is insufficient as a diagnostic tool, its integration with clinical and anthropometric measures such as BMI and maternal age may enhance predictive accuracy. Further prospective studies involving larger and more diverse populations are needed to validate these findings and to establish standardized cut-off values before clinical implementation can be recommended.

## Figures and Tables

**Table 1:** Demographic and clinical characteristics.

Characteristic	With Gestational Diabetes (*n* = 160) (Mean ± SD)	Without Gestational Diabetes (*n* = 160) (Mean ± SD)	*p*-Value
Age (years)	30.1 ± 4.8	28.5 ± 4.2	0.02
BMI (kg/m^2^)	27.5 ± 3.8	24.3 ± 3.1	<0.001
Blood Glucose (mg/dL)	95.6 ± 12.3	85.2 ± 10.5	<0.001
HOMA-IR	2.9 ± 1.1	1.8 ± 0.7	<0.001
Insulinemia (µU/mL)	12.4 ± 4.5	8.5 ± 3.2	<0.001
HOMA-β	180.5 ± 60.7	150.2 ± 50.3	0.01
Blood Pressure (mmHg)	120/80	110/70	0.03

**Table 2:** Pearson correlation coefficients (r) between HOMA-IR and clinical/anthropometric variables.

Variable	Pearson Correlation Coefficients (r)	*p*-Values
Fasting Glucose	0.65	<0.001
Fasting Insulin	0.85	<0.001
HOMA-BETA%	0.50	0.002
BMI (Body Mass Index)	0.60	<0.001
Age	0.30	0.04
Blood Pressure (BP) (mmHg)	0.25	0.05

Note: All coefficients represent bivariate correlations with HOMA-IR as the dependent variable. Variables include fasting glucose, insulin levels, HOMA-β, body mass index (BMI), blood pressure, and maternal age. Significance threshold: *p* < 0.01.

**Table 3:** Logistic regression analysis for predictors of gestational diabetes.

Variable	Odds Ratio (OR)	95% Confidence Interval	*p*-Value
HOMA-IR	2.4	1.6–3.5	<0.001
BMI (kg/m^2^)	1.2	1.1–1.4	0.01
Age (years)	1.1	1.0–1.2	0.05

**Table 4:** Distribution of GDM by cumulative metabolic risk score.

Metabolic Score (0–4)	Number of Patients (*n*)	GDM Cases (*n*)	Non-GDM Cases (*n*)	GDM Prevalence (%)
0	50	5	45	10%
1	60	15	45	25%
2	70	35	35	50%
3	80	60	20	75%
4	60	55	5	92%

## Data Availability

The original contributions presented in this study are included in the article. Further inquiries can be directed to the corresponding authors.
